# Is alcohol consumption a risk factor for prostate cancer? A systematic review and meta–analysis

**DOI:** 10.1186/s12885-016-2891-z

**Published:** 2016-11-15

**Authors:** Jinhui Zhao, Tim Stockwell, Audra Roemer, Tanya Chikritzhs

**Affiliations:** 1 Centre for Addictions Research of British Columbia, University of Victoria, PO Box 1700 STN CSC, Victoria, BC V8Y 2E4 Canada; 2 Department of Psychology, University of Victoria, PO Box 1700 STN CSC, Victoria, BC V8Y 2E4 Canada; 3 National Drug Research Institute, Curtin University, GPO box U1987, Perth, 6845 WA Australia

**Keywords:** Prostate cancer, Alcohol, Meta–analysis, Misclassification error

## Abstract

**Background:**

Research on a possible causal association between alcohol consumption and risk of prostate cancer is inconclusive. Recent studies on associations between alcohol consumption and other health outcomes suggest these are influenced by drinker misclassification errors and other study quality characteristics. The influence of these factors on estimates of the relationship between alcohol consumption and prostate cancer has not been previously investigated.

**Methods:**

PubMed and Web of Science searches were made for case–control and cohort studies of alcohol consumption and prostate cancer morbidity and mortality (ICD–10: C61) up to December 2014. Studies were coded for drinker misclassification errors, quality of alcohol measures, extent of control for confounding and other study characteristics. Mixed models were used to estimate relative risk (RR) of morbidity or mortality from prostate cancer due to alcohol consumption with study level controls for selection bias and confounding.

**Results:**

A total of 340 studies were identified of which 27 satisfied inclusion criteria providing 126 estimates for different alcohol exposures. Adjusted RR estimates indicated a significantly increased risk of prostate cancer among low (RR = 1.08, *P* < 0.001), medium (RR = 1.07, *P* < 0.01), high (RR = 1.14, *P* < 0.001) and higher (RR = 1.18, *P* < 0.001) volume drinkers compared to abstainers. There was a significant dose–response relationship for current drinkers (P_trend_ < 0.01). Studies free from misclassification errors produced the highest risk estimates for drinkers versus abstainers in adjusted models (RR = 1.22, *P* < 0.05).

**Conclusion:**

Our study finds, for the first time, a significant dose–response relationship between level of alcohol intake and risk of prostate cancer starting with low volume consumption (>1.3, <24 g per day). This relationship is stronger in the relatively few studies free of former drinker misclassification error. Given the high prevalence of prostate cancer in the developed world, the public health implications of these findings are significant. Prostate cancer may need to be incorporated into future estimates of the burden of disease alongside other cancers (e.g. breast, oesophagus, colon, liver) and be integrated into public health strategies for reducing alcohol related disease.

**Electronic supplementary material:**

The online version of this article (doi:10.1186/s12885-016-2891-z) contains supplementary material, which is available to authorized users.

## Background

Prostate cancer is the development of cancer in the prostate, a walnut–sized gland in men that surrounds the top of the urethra and which produces seminal fluid [1]. Its growth and functions are controlled by male hormones such as testosterone. Prostate cancer is the second most common cancer in men worldwide. Around 1.1 million cases were recorded in 2012, accounting for 15% of all new cases of cancer in men [2]. It is most commonly diagnosed in high–income countries, where screening is common. It is the fifth most common cause of cancer death in men worldwide. Therefore prostate cancer as a chronic disease has become an important public health concern.

The risk factors for prostate cancer that can be considered established include age, race/ethnicity and family history [3]. Many observational studies have investigated alcohol consumption as a risk factor for prostate cancer. Conclusions from these studies and of reviews have been conflicting with some finding increased risk of prostate cancer [4–6], or decreased risk [7] and others finding no relationship [8–13]. While many unidentified and uncontrolled factors or biases may have confounded the relationships of interest in these studies, an additional concern is that former and occasional drinkers may be misclassified into the abstaining reference group. Previous studies have showed that such misclassification can bias estimates of health risks from alcohol use, for example, underestimating risks from low–volume drinking [14–19]. Former and occasional drinkers may include people who have stopped or reduced their drinking as they aged and experienced declining health [16, 20]. Thus including former and occasional drinkers can bias the abstaining reference group towards reduced health and by comparison, reduce estimated disease risk from drinking.

Over the past few decades there have been several reviews and meta–analyses conducted to examine the association of prostate cancer with alcohol consumption [7, 8, 13, 21–25]. Early reviews by Longnecker [13] and Morton et al. [8] both concluded there was no relationship. Breslow and Weed [24] reviewed 32 studies of which only six reported significant associations between risk of prostate cancer and alcohol consumption. Dennis [22] conducted a meta–analysis on six cohort and 27 case–control studies, finding no overall association between prostate cancer and any alcohol consumption. However, when they examined 15 studies in which the relative risks (RR) for drinking levels were available, they found that three or more drinks per day increased the risk of prostate cancer. Dagnelie et al. (2004) [7] reviewed nine studies on prostate cancer and total alcohol consumption and found that six studies reported no association, two reported an increased risk and one a decreased risk. A meta–analysis by Bagnardi et al. [25] found a small but significantly increased risk for men drinking more than 50 g/day of alcohol, with a slightly higher risk for men consuming more than 100 g/day but there was no significant dose–response relation. This meta–analysis was the first to consider potential confounding, between–study variation and modifying effects of tobacco smoking but did not control for drinker misclassification errors. A meta–analysis by Fillmore et al. [21] found a significant relationship between prostate cancer and heavy alcohol use after controlling for the effects of median age of study populations, design and between–study variation. Rota et al. [23] found a significantly higher RR of prostate cancer for any drinking, light (≤1 drink/day) and moderate drinking (>1, <4 drink/day) versus abstaining/occasional drinking but the analysis found no significant relationship with heavy drinking (≥4 drinks/day) and did not consider the potential effects of misclassification. In summary, more recent reviews and meta–analyses have been more likely to find positive associations but none have adequately considered the effects of confounding and bias, including potential biases caused by misclassification of former and occasional drinkers in the abstainer reference groups.

The objectives of the present meta–analysis were: (i) to investigate the relationship between prostate cancer and alcohol consumption; and (ii) to examine whether estimates of this relationship may have been biased by drinker misclassification errors and other study characteristics.

## Methods

### Inclusion and exclusion criteria

The criteria for inclusion were: (i) case–control and cohort studies evaluating the relationship between alcohol consumption and prostate cancer; (ii) original articles published in English up till December 2014; (iii) articles that reported findings in odds ratio, hazard ratio, incidence ratio or standardized mortality ratio; and (iv) articles reporting at least three levels of alcohol consumption with drinking amounts, including the reference level. Articles with no abstainer group or a lowest drinking level greater than 0.33g/d were excluded. Additionally, studies reporting total alcohol consumption were included while studies based on consumption of specific beverages only such as wine, whiskey, vodka, sake or hard liquors were excluded. When the results of the study were published more than once or if the same dataset was used multiple times, only the most recent or more complete data were included in analyses. The primary outcomes of interest were mortality and/or morbidity from prostate cancer (ICD–9: 185 or ICD–10: C61) [26].

While published and peer reviewed cohort or case–control studies were included in the review, all other article types including narrative reviews, letters, editorials, commentaries, unpublished manuscripts, dissertations, government reports, books and book chapters, conference proceedings, meeting abstracts, lectures and address, and consensus development statement including guideline statements, were excluded.

### Search strategy

The systematic review follows the Preferred Reporting Items for Systematic Reviews and Meta–Analyses (PRISMA) guidelines [27]. We identified all potentially relevant articles by searching Pubmed and Web of Science, through reference list cross–checking including those of previous meta–analyses and incorporating publications up to 31 December 2014. Hand searches of cited references in the selected articles, reviews and meta–analysis published on the same topic were also performed. The following MESH terms and text words were used: (“prostatic neoplasms” OR (“prostate” AND “neoplasms”) OR “prostate cancer “OR (“prostate” AND “Cancer”)) AND (“alcohol” OR (alcohol drinking) OR “alcohol consumption” OR “alcohol intake” OR (“alcohol” AND “consumption”)).

### Study selection

**Fig. 1:**
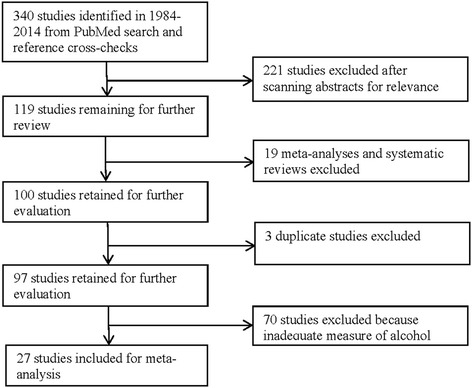
Flowchart of summarizing systematic review of studies of prostate cancer morbidity or mortality and alcohol consumption from literature search to inclusion in meta–analysis

Two reviewers trained and supervised by the PI read the titles and/or abstracts of all the citations retrieved from the electronic database searches and removed all citations that were clearly not related to studies of the relationship between prostate cancer and alcohol consumption. The screening further involved abstract review. Full–text articles were obtained for all abstracts except for those that clearly did not meet eligibility criteria. The investigators were consulted in the event of any disagreement. Two of the investigators independently evaluated all studies selected for inclusion. The initial search identified a total of 340 studies of which 27 studies [4–6, 9, 11, 12, 28–48] satisfied the criteria for the meta–analysis after removing 313 records for reasons identified in Fig. 1.

### Data extraction

Two reviewers independently reviewed all eligible papers to extract and code data from all studies fulfilling the inclusion criteria, and any disagreements were resolved by discussion with the investigators. Each study was coded with reference to a standardized code–book (available from authors on request) and under the supervision of investigators. The coding of all variables in the meta–dataset was double–checked by the first two authors. The data to be extracted were: (1) outcome, mortality or morbidity of prostate cancer; (2) measures of alcohol consumption; (3) study characteristics; (4) types of misclassification error of alcohol measure; and (5) controlled variables in individual studies.

A multitude of different approaches are used for assessing alcohol consumption in this literature [49]. Problematic approaches include assessing some beverage types and not others, assessing quantity consumed on a drinking day but not frequency, assessing consumption over very short time periods (e.g. two days) and assessing frequency but not quantity of consumption. We coded alcohol measurement as ‘adequate’ if both quantity and frequency of consumption was assessed for all alcoholic beverages and for a period of at least one week.

The primary exposure variable was level of daily alcohol consumption in grams of ethanol assessed at baseline and compared with a reference group of variously defined “non–drinkers” or “abstainers”. When studies did not define the grams of alcohol per unit or drink, we used 8 g/unit for the UK; 10 g/drink for Australia, Austria, France, Greece, Hungary, Ireland, Netherlands, New Zealand, Poland, Spain, Sweden; 11 g/drink for Finland; 12 g/drink for Denmark, Germany, Italy, South Africa and Switzerland; 13.45 g/drink for Canada; 14 g/drink for US; 12.5 g/drink for China, 19.75 g/drink for Japan and 12 g/drink for other countries [50, 51]. We converted alcohol intake into grams per day using the mid–points of reported categories to estimate mean values. Following practice in other meta–analyses involving self–reported alcohol consumption, the open–ended top categories (e.g. 6+ drinks/day) were coded by adding three–quarters of the range of the next lowest category to the lower bound (e.g. if 3 to 5 drinks this would be 6 + (5–3)*0.75 = 7.5) [52]. It is necessary to make some higher estimate than the lowest level possible for these open–ended categories with no fixed upper level (e.g., 7.5 in this case instead of 6 for 6+ drinks). We employed predetermined definitions of “low–volume” drinking (up to 20g ethanol per day) based on Australian NHMRC low risk drinking guidelines [53]. This was operationalised as up to 24 g per day given that respondents in the studies reported whole drinks or units rather than grams i.e. 24g per day is closer to two than three 10g standard drinks per day. All data extracted from individual studies and analyzed during this study can be found in Additional file 1.

Studies were classified according to the presence or absence of two types of potential abstainer group bias: (i) including former drinkers and/or (ii) including occasional drinkers in the abstainer reference category. Studies were coded as having former drinker bias if a) results were not reported separately for former drinkers and b) there was no mention of removing former drinkers from the abstainer reference group. Following Fillmore et al. [16], lifetime abstention was strictly defined as zero consumption and did not include studies with any level of occasional lifetime or past year drinking (e.g. less than 12 drinks or “rarely” or “hardly ever” drinking). Our rationale for this strict criterion was that self–reported infrequent drinkers have been shown to greatly underreport their personal consumption [54, 55]. Studies were coded as having occasional drinker bias if a) results were not reported separately for occasional drinkers and b) frequency of drinking was assessed for a “usual” period or over less than 30 days. The rationale here is that if a person reports “usually” not drinking over the course of a month, persons drinking less than monthly may still be occasional drinkers. When a study used occasional drinkers as the reference category and risk for abstainers was independently assessed, the risk values were recalculated using the abstainer category as the reference group [16].

### Strategy for data analysis

Where studies only reported mortality or incidence rates, these were converted to RR estimates [56]. Otherwise hazard ratios in cohort studies and odds ratio estimates in case–control studies were entered as observations of the estimated risk relationships for meta–analysis. When the odds ratios (OR as RR estimates) are estimated using logistic regression models in a case–control study, the OR tends to overestimate RR when it is more than one and to underestimate RR when it is less than one if the outcome becomes more frequent [57]. Therefore, the formula below was used to correct the adjusted OR and its 95% CIs obtained from logistic regression in studies and derive an estimate of an association that better represents the true RR [57].

$$ RR=\frac{OR}{\left(1-{P}_0\right)+\left({P}_0\times OR\right)}, $$

where *RR* is relative risk, *OR* is odds ratio and *P*_0_ is the incidence of outcome of interest in the non–exposed group.

Publication bias was assessed through visual inspection of the funnel plot of log–RR of morbidity or mortality of prostate cancer due to alcohol consumption against the inverse standard error of log–RR [56] and Egger’s linear regression method [58]. We plotted a forest graph to examine how the RR estimate for any drinking in one study is different from others [56]. We also assessed between–study heterogeneity of RRs overall and by drinking groups using Cochran’s Q [59] and the I^2^ statistic [60]. As no heterogeneity was detected, fixed effects models were used to obtain the summarized RR estimates [56]. We also conducted sensitivity tests using random effects models, but patterns of results were very similar and are not reported here.

We used the fixed effects models to estimate the weighted RRs of prostate cancer for any alcohol use and by drinking groups while adjusting for the potential effects of study–level covariates [56, 61–63]. Drinking level in each study group was examined in terms of pre–defined specific consumption levels. Drinking categories were defined and reclassified as: (1) lifetime occasional drinkers (0.02–0.33 g/day); (2) former drinkers now completely abstaining; (3) current occasional drinkers, up to one drink per week (<1.30 g per day); (4) low volume drinkers, up to 2 drinks or 1.30–24 g per day; (5) medium volume, up to 4 drinks or 25–44g per day; (6) high volume drinkers, up to 6 drinks or 25–64g per day; and (7) higher volume drinkers, 6 drinks or 65g or more per day. All studies had an open–ended heavier drinking group, i.e., with no upper limit of quantity consumed per day for responses accepted as valid. We investigated the dose–response relationship between the RR and alcohol consumption for those who drank one drink or more per week using the midpoint of each exposure category using *t*-test in multivariate linear regression analysis [56].

We investigated the potential modification and confounding effects of study–level covariates using bivariate analysis of RR of prostate cancer morbidity or mortality and any alcohol consumption [64]. According to the availability of the data from 27 included studies, the following study characteristics were investigated: (1) study designs which included cohort study, population–based case–control study and hospital–based case–control study; (2) outcomes, i.e., morbidity or mortality of prostate cancer; (3) adequacy of drinking measurement method defined as whether both quantity and frequency of total alcohol consumption was assessed for at least one week; (4) mean or median age of individual study populations at baseline; (5) year at baseline, if recruited over a number of years then take midpoint; (6) whether subjects with a history of cancer were excluded at baseline or prior to randomization (yes, no or unknown); (7) presence of misclassification errors, i.e., including both former and occasional drinkers, only former drinkers, only occasional drinkers or neither former nor occasional drinkers in the abstaining reference group; (8) whether or not the study and control for social status (yes or no) using income or occupation measures; (9) whether or not a study controlled for racial identity or country of origin (yes or no); (10) whether or not a study control for smoking status (yes or no); (11) whether or not a study was conducted in US. We made stratified RR estimates for studies with different values for these characteristics and also examined the differences in the RR estimates between these same subgroups of studies [64].

The covariates above were selected for control in multivariate regression analyses on empirical grounds based on the *P*–value of bivariate tests of the log–RR of each covariate, and correlations with other covariates. Using all 27 studies, any variable whose bivariate test had a *P*–value <0.10 was considered as a candidate for the multivariate regression analyses of the log–RR of prostate cancer morbidity or mortality [65, 66]. If two or more covariates were moderately to highly correlated (coefficient > 0.30), the one with lowest *P*–value from the bivariate test was included in the multivariate regression analyses. Abstainer bias was the main interest of the present study and thus its potential confounding effect was adjusted for in the pooled analysis (Table 3) and further examined in the stratified analysis (Table 4). On the basis of these criteria, two other covariates were included in the analyses: (i) whether or not the study was conducted in the US and (ii) whether smoking was controlled in the individual studies (Tables 3 and 4). Although the study design variable was not selected as a controlled covariate in the final models using bivariate analysis, the study design was a concern as these were unevenly distributed across the studies with different abstainer biases and the RR estimates were slightly different in case-control studies from cohort studies [23]. We still examined the potential effect of the design variable by performing a sensitivity analysis by including and excluding it in multivariate regression analyses (Tables 3 and 4). However, the estimates remained unchanged. We also conducted a correlation analysis of the study design variable and other selected covariates. The design variable was highly correlated with the abstainer bias variable (the coefficient = 0.48 and *P* < 0.001) and it was not included in the final models.

In multivariate regression analysis, the dependent variable was the natural log of the RR estimated using the rate ratio, hazard ratio or odds ratio of each drinking group in relation to the abstainer category. All analyses were weighted by the inverse of the estimated variance of the natural log RR. Variance was estimated from reported standard errors or confidence intervals. The weights for each individual study were created using the inverse variance weight scheme used in fixed regression analysis in order to obtain maximum precision for the main results of the meta–analysis [56] and such analyses may adjust for confounding among the characteristics [63].

Studies with large or small estimates and/or variance can be highly influential. Univariate analysis [56, 67, 68] was performed to identify outliers. If a particular RR was more than twice the standard deviation of the RR estimates by drinking groups it was considered to be an outlier; five risk estimates were identified as outliers among 126 risk estimates. Sensitivity analyses were run after excluding outliers but no substantial changes in the risk estimates resulted [56]. A sensitivity analysis was also run after excluding one study by Putnam et al. [41] with markedly higher risk estimates but, again, the estimates remained unchanged. There was also no substantial effect on the RR estimates when each of other studies were excluded or included.

All significance tests assumed two–tailed *P* values or 95% CIs. All statistical analyses were performed using SAS 9.3 and the SAS PROC MIXED procedure was used to model the log–transformed RR [69].

### Role of the funding sources

The study funders had no role in study design, data collection, analysis or interpretation, report preparation and the decision to publish. All authors had full access to all the data and had final responsibility for the decision to submit for publication.

## Results

**Fig. 2:**
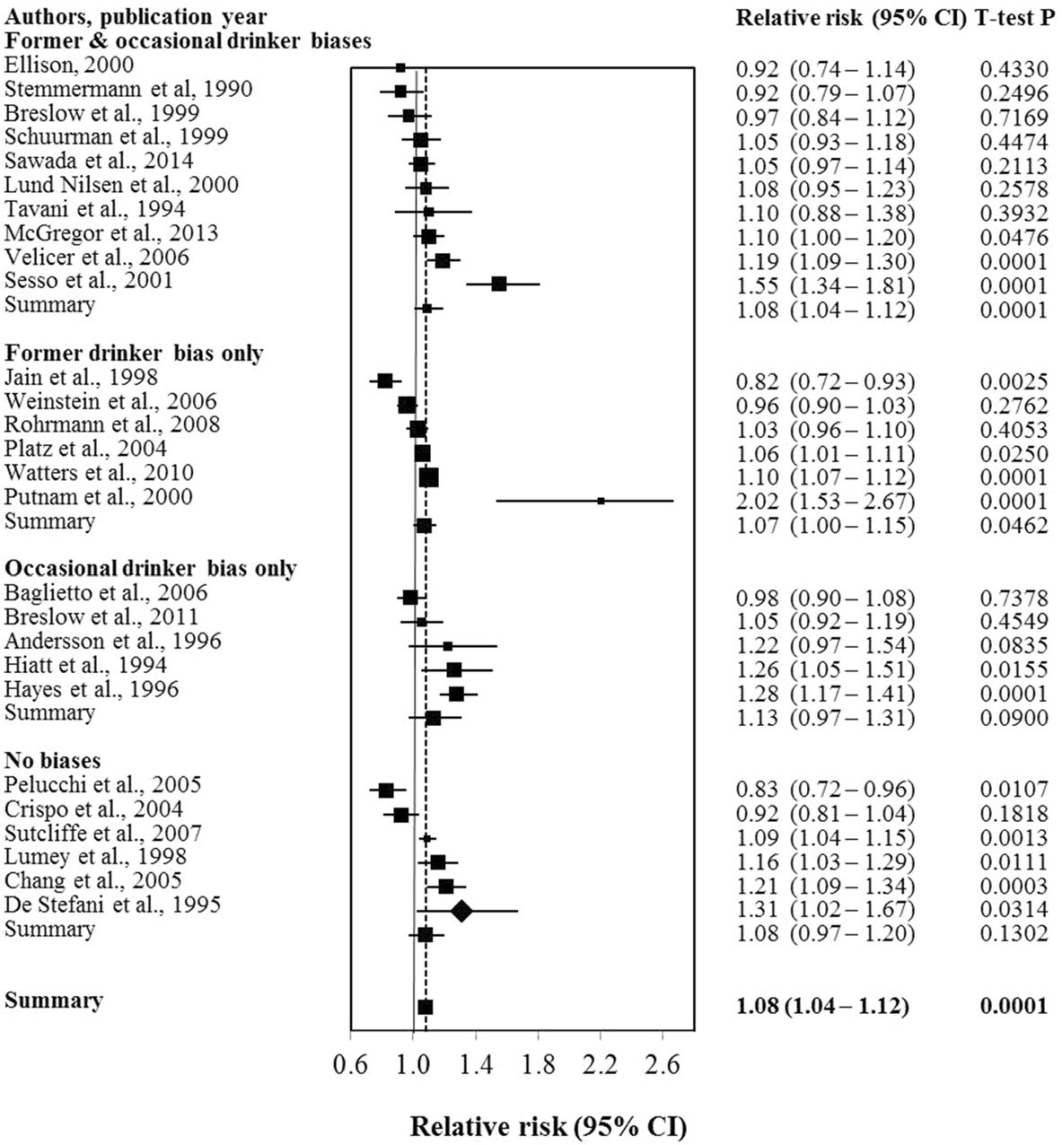
Relative risk (95% CI) of prostate cancer morbidity or mortality for any alcohol consumption versus “abstaining” in 27 studies

There were 126 risk estimates available for different alcohol exposures across the 27 selected studies. Table 1 presents the basic characteristics of these studies including covariates included in individual studies. As shown in Table 1, there were 16 prospective and one retrospective cohort studies, five hospital–based case–control and five population–based case–control studies. A forest plot (see Fig. 2) displays the weighted RR estimates for the risk of prostate cancer associated with any level of drinking versus “abstaining” reported in individual studies, grouped according to the type of misclassification error present. A visual inspection of Fig. 2 indicates considerable cross–study variation in estimates.

**Table 1:** Characteristics of 27 included studies for meta–analysis on prostate cancer and alcohol consumption

Author	Study country	Cases/N^a^	Outcome^b^	Design^c^	Age range	Follow–up yrs	Covariates assess^d^
Studies with both biases							
Stemmermann, [11]	US	227/8006	M/M	P–cohort	46–65	25.5	1,4
Tavani et al., [12]	Italy	281/880	Morb	Hos–CC	25–79	u/a	1,2,4,5
Breslow et al., [30]	US	252/5766	M/M	P–cohort	25–75	17.0	1,2,3
Schuurman et al., [44]	Netherlands	680/58279	Morb	P–cohort	55–69	6.3	1,2
Lund Nilsen et, [37]	Norway	644/22895	M/M	P–cohort	40–99	9.3	1
Ellison, [34]	Canada	145/3400	M/M	R–cohort	50–84	23.0	1
Sesso et al., [4]	US	366/7612	M/M	P–cohort	30–68	5.0	1,4,5,6
Velicer et al., [46]	US	816/34565	Morb	P–cohort	50–76	4.0	1
McGregor et al., [38]	Canada	947/1986	Morb	Pop–CC	–79	u/a	1
Sawada et al., [43]	Japan	913/48218	Morb	P–cohort	40–79	16.0	1,4,5
Studies with former drinker bias only							
Jain et al., [35]	Canada	617/1254	Morb	Pop–CC	48–92	u/a	1,4
Putnam et al., [41]	US	101/1572	M/M	P–cohort	40–86	9.0	1
Platz et al., [40]	US	2479/47843	M/M	P–cohort	40–75	12.0	1,3,5,6
Weinstein et al., [47]	Finland	1270/27111	M/M	P–cohort	50–69	17.0	1,7
Rohrmann et al., [42]	Europe^e^	2655/142647	M/M	P–cohort	40–65	8.7	4,5
Watters et al., [5]	US	17227/294707	M/M	P–cohort	50–71	7.0	1,2,3,4,5,6
Studies with occasional drinker bias only							
Hiatt et al., [9]	US	238/43432	Morb	P–cohort	30–99	4.6	1,2,3
Andersson et al., [28]	Sweden	256/508	Morb	Pop–CC	–79	u/a	1
Hayes et al., [6]	US	981/2296	Morb	Pop–CC	40–79	u/a	1,3
Baglietto et al., [29]	Australia	732/16872	M/M	P–cohort	27–70	10.3	1
Breslow et al., [48]	US	8362/323354	Mort	P–cohort	18–99	8.3	1,2,3,5
Studies with neither abstainer bias							
De Stefani et al., [33]	Uruguay	156/458	Morb	Hos–CC	40–89	u/a	1,2,4
Lumey et al., [36]	US	699/2740	Morb	Hos–CC	36–81	u/a	1,2,3
Crispo et al., [32]	Italy	2663/4114	Morb	Hos–CC	46–74	u/a	1,2,5,6
Chang et al., [31]	Sweden	1499/2629	Morb	Pop–CC	45–79	u/a	1
Pelucchi et al., [39]	Italy	1294/2745	Morb	Hos–CC	46–79	u/a	1
Sutcliffe et al., [45]	US	3348/45433	M/M	P–cohort	40–75	16.0	1,3,5,6

Note: ^a^ *N* = cases + controls in a case–control study. ^b^M/M = mortality and morbidity, Morb = morbidity and Mort = mortality. ^c^P–cohort = prospective cohort, R–cohort = retrospective cohort, Pop–CC = population–based case–control, Hos–CC = hospital–based case–control. ^d^1: age; 2: social status; 3: race; 4: smoking status; 5: body mass index; 6: exercise. ^e^10 European countries: Denmark, France, Germany, Great Britain, Greece, Italy, The Netherlands, Norway, Spain and Sweden

**Fig. 3:**
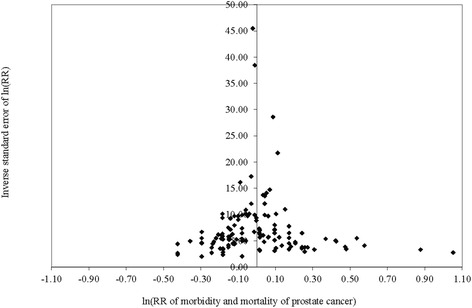
Funnel plot of relative risk (ln(RR)) of prostate cancer morbidity or mortality due to alcohol consumption against inverse standard error of ln (RR)

Table 2 presents unadjusted mean RR estimates of prostate cancer morbidity or mortality by level of alcohol consumption with tests of publication bias and heterogeneity. Figure 3 provides a funnel plot showing the log–RRs and their inverse standard error from which there was no indication of publication bias as the plot is reasonably symmetrical. No significant publication bias was detected using the Egger’s regression either for the pooled data or the individual drinking categories data (*P* > 0.05 for each drinking category). Similarly, there was no significant heterogeneity detected using the Q statistic in either the pooled or individual drinking category estimates (*P* > 0.05 in each case). Compared to the “abstainers” (a heterogeneous group defined differently in different studies due to presence or absence of misclassification errors), being a drinker at any level was associated with increased risk of prostate cancer (RR = 1.08, 95% CI: 1.04–1.12, *P* = 0.0033). Risk of prostate cancer was significantly raised for low (RR = 1.09, *P* = 0.0031) and higher volume drinkers (RR = 1.15, *P* = 0.0336) but not other drinking categories. In unadjusted analysis, a significant dose–response relationship in the RR was observed among active drinkers (t–test statistic = 3.42, *P* = 0.0009).

**Table 2:** Unadjusted mean RR estimates of prostate cancer morbidity or mortality for different categories of drinkers compared with ‘abstainers’ (*N* = 27 studies and 126 observations) with tests of publication bias and heterogeneity

Drinking categories	N/n^a^	Unadjusted mean RR		Egger’s regression for publication bias		Test for heterogeneity	
		RR (95 % CI)	*t*–test *P*	Coefficient	*t*–test *P*	Q statistic *P*	I^2^ (%, 95 % CI)
Abstainer		1.00					
Former drinker	7/13	1.04 (0.92 – 1.19)	0.5155	–1.33	0.2274	>0.05	1.00 (0.00 – 56.59)
Occasional (<1.30 g/day)	6/7	1.02 (0.86 – 1.21)	0.8292	+1.63	0.0542	>0.05	1.00 (0.00 – 70.81)
Low volume (1.30– < 25 g/day)	27/62	1.09 (1.03 – 1.16)	0.0031	+0.13	0.5558	>0.05	10.66 (0.00 – 64.80)
Medium volume (25– < 45 g/day)	18/20	1.03 (0.93 – 1.14)	0.6046	–0.26	0.4287	>0.05	1.00 (0.00 – 62.37)
High volume (45– < 65 g/day)	10/11	1.13 (0.98 – 1.30)	0.0935	–0.32	0.5125	>0.05	13.38 (0.00 – 53.90)
Higher volume (65+ g/day)	9/13	1.15 (1.01 – 1.13)	0.0336	–0.24	0.6353	>0.05	19.94 (0.00 – 57.85)
Any drinking	27/126	1.08 (1.04 – 1.12)	0.0033	–0.04	0.7727	>0.05	16.42 (0.00 – 33.73)

Note: ^a^ *N* = Number of studies and *n* = Number of risk estimates

**Fig. 4:**
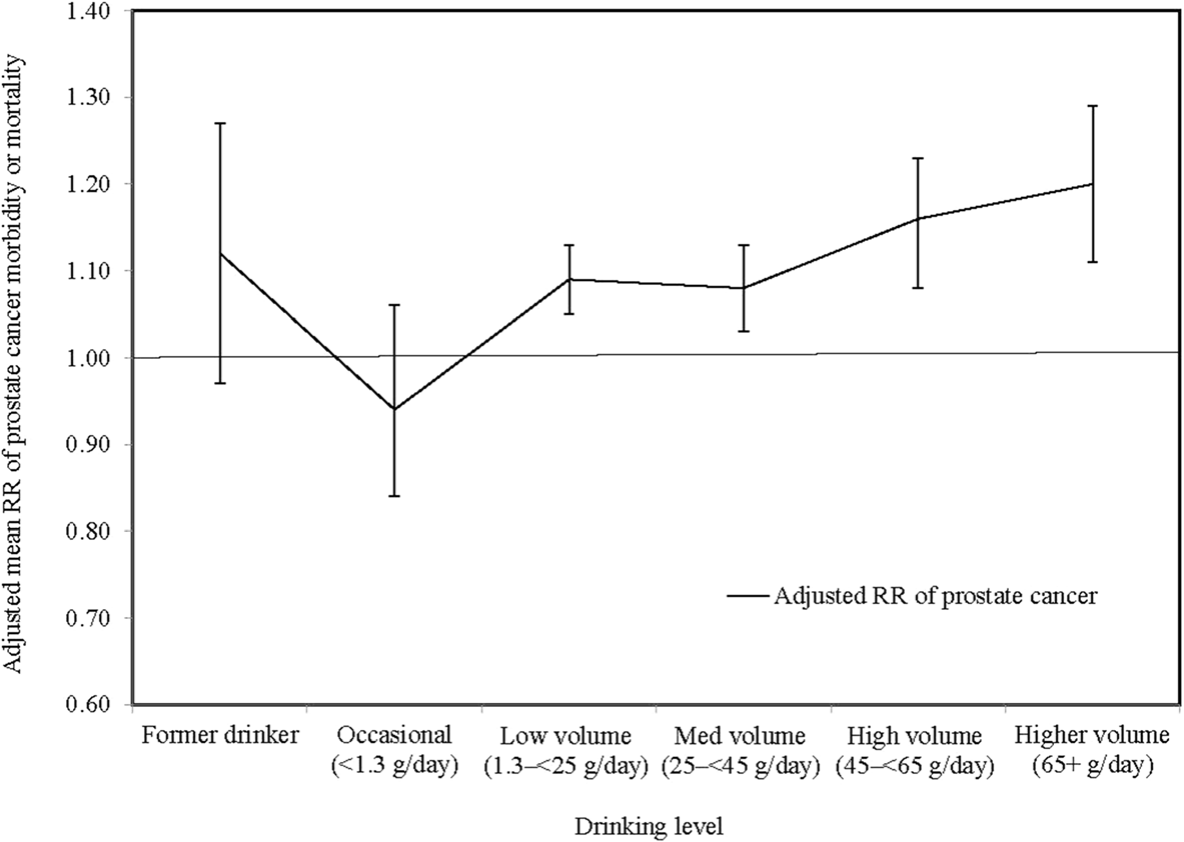
Adjusted mean relative risk (RR) of prostate cancer morbidity or mortality due to alcohol consumption

We next examined whether study characteristics either significantly modified or potentially confounded the risk relationships between alcohol consumption and prostate cancer morbidity or mortality outcomes. The weighted RR estimate for any drinking versus non–drinking is significantly higher for US than non–US studies (t–test *P* = 0.0005) but not significant for low volume drinking versus non–drinking (t–test *P* = 0.1432) (see Additional file 2. Weighted RR estimates according to study characteristics”). When further investigating whether the US vs. non–US variable was a modifier, the interaction term in the model was not statistically significant (*P* = 0.9580) and so meta-analyses are presented on a pool of both US and non-US studies. When tests with low volume alcohol exposure alone were conducted (see Additional file 2) a borderline modification effect with the misclassification error variable was evident (*P* = 0.0767) for the comparison between studies free of misclassification errors and those with just former drinker error. Two other variables were identified in bivariate analyses as potential confounders of the risk relationship between alcohol consumption and prostate cancer morbidity or mortality: (i) whether the US–non–US study (*P* = 0.0019) and (ii) whether a study controlled for smoking status (*P* = 0.0838). The misclassification error variable was included as covariates in the pooled (un–stratified) multivariate regression analysis given previous research highlighting their importance. Table 3 presents weighted only, partially adjusted and fully adjusted mean RR estimates of morbidity or mortality due to prostate cancer for different drinking categories. The weighted RR estimates without further adjustment were significantly higher for low, medium, high and higher volume drinkers than abstainers. After further adjusting for the confounding effect of drinker biases (partially adjusted), the RR estimates increased. After further adjusting for US-non-US study and controlled smoking (fully adjusted), there was a statistically significantly increased risk of prostate cancer for low (adjusted RR = 1.08, 95% CI = 1.04–1.11 and t–test *P* = 0.0001), medium (adjusted RR = 1.07, 95% CI = 1.02–1.12 and t–test *P* = 0.0041), high (adjusted RR = 1.14, 95% CI = 1.08–1.22 and t–test *P* = 0.0001) and higher volume drinkers (adjusted RR = 1.18, 95% CI = 1.10–1.27 and t–test *P* = 0.0001). There was also still a significant dose–response relation between risk of prostate cancer and alcohol consumption for current drinkers in adjusted analysis (Fully adjusted model, t–test statistic = 2.79, P_trend_ = 0.0063). Figure 4 presents the adjusted RRs for different drinking levels.

**Table 3:** Adjusted mean RR estimates of prostate cancer morbidity or mortality for different categories of drinkers compared with abstainers (*N* = 27 studies and 126 risk estimates)

Drinking categories	N/n^a^	Weighted Mean RR^b^			Partially adjusted mean RR^c^			Fully adjusted mean RR^d^		
		RR & 95 % CI		*t*–test *P*	RR & 95 % CI		*t*–test *P*	RR & 95 % CI		*t*–test *P*
Abstainer		1.00			1.00			1.00		
Former drinker	7/13	1.14	0.99 – 1.30	0.0642	1.12	0.98 – 1.29	0.0990	1.10	0.97 – 1.25	0.1348
Occasional (<1.30 g/day)	6/7	0.97	0.86 – 1.10	0.6126	0.96	0.85 – 1.08	0.5265	0.95	0.85 – 1.06	0.3263
Low volume (1.30– < 25 g/day)	27/62	1.07	1.04 – 1.10	0.0001	1.08	1.05 – 1.12	0.0001	1.08	1.04 – 1.11	0.0001
Medium volume (25– < 45 g/day)	18/20	1.06	1.02 – 1.11	0.0068	1.08	1.03 – 1.13	0.0023	1.07	1.02 – 1.12	0.0041
High volume (45– < 65 g/day)	10/11	1.15	1.08 – 1.22	0.0001	1.17	1.09 – 1.25	0.0001	1.14	1.08 – 1.22	0.0001
Higher volume (65+ g/day)	9/13	1.18	1.09 – 1.27	0.0001	1.20	1.11 – 1.29	0.0001	1.18	1.10 – 1.27	0.0001
Any drinking	27/126	1.08	1.04 – 1.12	0.0039	1.10	1.02 – 1.18	0.0214	1.08	1.01 – 1.17	0.0364

Note: ^a^ *N* = Number of studies and *n* = Number of risk estimates. ^b^Weighted using the inverse of variance of natural log–RR. ^c^Weighted RR estimates adjusted for both former and occasional drinker biases. ^d^Weighted RR estimates adjusted for between–study variation, both former and occasional drinker biases, US/non–US study and control for smoking status in individual studies

Given the previous literature indicating the potential for misclassification errors to bias risk estimates, visual inspection of the Fig. 2 and the borderline evidence for effect modification in Additional file 2, we also present results stratified by type of misclassification errors detected in Table 4. These show substantially different estimates according to the presence or absence of different misclassification errors with studies free from errors having the highest RR estimate for low volume drinkers (RR = 1.23, 95% CI: 1.05–1.45, *P* = 0.0143) and those with only former drinker bias having the lowest (RR = 1.01, 95% CI: 0.96–1.06, *P* = 0.6901). A similar pattern of results was evident for higher levels of alcohol consumption and, also, for estimates of prostate cancer risk with any level of current alcohol consumption for which only the error–free studies show a significant risk for drinking regardless of whether adjustment is made whether studies controlled for US-non-US study or smoking. Sensitivity analysis found that inclusion or exclusion of the study design variable in the models made no difference to the estimates. These results are basically consistent with the pooled analysis in suggesting an increased risk even for low volume drinking but also indicate the importance of misclassification errors as a potential cause of bias. In particular, inclusion of former drinkers in the abstaining reference group appears to reduce the risk estimates.

**Table 4:** Adjusted mean RR estimates of prostate cancer morbidity or mortality for different categories of drinkers compared with ‘abstainers’ by misclassification errors

Drinking categories	N/n^a^	Weighted for mean RR			Adjusted for mean RR^b^		
		RR	95 % CI	*t*–test *P*	RR	95 % CI	*t*–test *P*
Former & occasional drinker biases							
Low–volume (1.30– < 25 g/day)	10/20	1.10	1.03 – 1.18	0.1499	1.11	1.03 – 1.19	0.0069
Medium–high volume (25+ g/day)	9/17	1.12	1.02 – 1.22	0.0152	1.13	1.04 – 1.24	0.0079
Any drinking (1.30+ g/day)	10/37	1.11	1.05 – 1.17	0.0273	1.12	1.02 – 1.23	0.0405
Former drinker bias only							
Low–volume (1.30– < 25 g/day)	6/16	1.05	1.00 – 1.10	0.0708	1.01	0.96 – 1.06	0.6901
Medium–high volume (25+ g/day)	5/10	1.10	1.05 – 1.16	0.0005	1.05	0.99 – 1.12	0.1136
Any drinking (1.30+ g/day)	6/26	1.07	0.84 – 1.37	0.1657	1.03	0.87 – 1.22	0.2760
Occasional drinker bias only							
Low–volume (1.30– < 25 g/day)	5/10	1.08	0.97 – 1.21	0.1411	1.06	0.95 – 1.18	0.2534
Medium–high volume (25+ g/day)	4/9	1.06	0.95 – 1.18	0.2674	1.01	0.89 – 1.15	0.8706
Any drinking (1.30+ g/day)	5/19	1.07	0.97 – 1.18	0.0715	1.04	0.83 – 1.29	0.2955
Neither former or occasional drinker biases							
Low–volume (1.30– < 25 g/day)	6/16	1.12	1.05 – 1.18	0.0008	1.23	1.05 – 1.45	0.0143
Medium–high volume (25+ g/day)	3/8	1.10	0.95 – 1.26	0.1798	1.20	1.00 – 1.43	0.0475
Any drinking (1.30+ g/day)	6/24	1.11	1.03 – 1.19	0.0359	1.22	1.07 – 1.38	0.0321

Note: ^a^ *N* = Number of studies and *n* = Number of risk estimates. ^b^Weighted using the inverse of variance and adjusted for US vs non–US study and control of smoking status in individual studies

## Discussion

Meta–analyses of cohort and case–control studies were conducted to investigate (i) the role of alcohol consumption as a potential risk factor for prostate cancer and, (ii) whether this relationship was significantly influenced by key study characteristics and potential biases, in particular according to whether former and/or occasional drinkers were misclassified as abstainers. Unique among published meta-analyses [21–23, 25], we report a significant dose response relationship to be observed with increasing risk of prostate cancer starting at low–level alcohol consumption (>1.33g and <25g ethanol/day) regardless of adjustment for study characteristics in pooled models of all 27 eligible studies. High (45– < 65 g/day) and higher (65+ g/day) volume drinkers had a significantly higher risk (RR = 1.14 and 1.18). Further, there was no significant heterogeneity in study estimates or evidence of publication bias. However, when analyses were stratified by whether or not studies misclassified former and/or occasional drinkers as abstainers, it was evident that former drinker bias reduced overall risk estimates to the extent that alcohol exposure at any level was no longer associated with significantly increased risk of prostate cancer. Out of 27 studies included, 16 contained former drinker bias, 15 occasional drinker bias only, six were free from both types of bias. It can be concluded that the common practice of combining former drinkers with abstainers in prospective studies of alcohol consumption and health biases risk estimates downwards and can lead to underestimation of the risks posed by low volume consumption. There was no indication that misclassifying occasional drinkers contributed to significant downward bias in risk estimates and, further, when estimates were made separately for occasional drinkers the RRs tended to be slightly lower. We conclude that the common practice of misclassifying former drinkers as abstainers, especially in older studies, has sometimes disguised a significant association between alcohol exposure and risk of prostate cancer.

Alcohol is a known carcinogen causing a variety of human cancers [70] via different biological pathways depending on the anatomical site. The evidence that alcoholic drinks are a cause of cancers of the mouth, pharynx, larynx, oesophagus, liver, colorectum and breast in women is compelling [25, 70, 71]. Alcoholic beverages are multicomponent mixtures containing several carcinogenic compounds such as ethanol, acetaldehyde, aflatoxins and ethyl carbamate [72] and all of these compounds may contribute to increase the risk of cancer due to alcohol consumption reported in observational studies. The biological mechanisms by which alcohol intake might increase the risk of prostate cancer are not fully understood but the main mechanisms are likely to include a genotoxic effect of acetaldehyde, the induction of microsomal cytochrome P450 2E1 (CYP2E1) and associated oxidative stress, increased estrogen concentration, a role as a solvent for tobacco carcinogens, changes in folate metabolism, and changes in DNA repair [73–75].

Several limitations with our meta–analysis must be acknowledged. Our meta–analysis was based on 27 studies including 126 risk estimates. This sample is relatively small when conducting multivariate regression to control for study level characteristics that might confound the relationship between prostate cancer and alcohol consumption. Furthermore, adjustment for study level characteristics such as whether smoking status was controlled is of course not as precise as controlling for this variable at the individual level within a study. Inevitably, uncontrolled confounding from unmeasured or imprecisely measured variables will be present both within and between studies. Control for smoking status, for example, can be done in many ways and some studies did not distinguish former smokers from lifetime non–smokers. Our analysis showed a statistically significantly higher risk of prostate cancer due to any drinking in the studies conducted in the US than in other countries. However, this effect disappeared when controls for other covariates such as drinker bias and between-study variation were introduced. This finding might be affected by the relatively small number of studies available for analysis and/or other unmeasured confounders and modifiers. The issue should be revisited in future meta-analyses when more studies are available. While there were relatively few studies available for analysis, the required number of subjects per variable for linear regression is much smaller than in logistic regression and a minimum of even two subjects per variable would not bias the estimate in linear regression analysis [76]. The great majority of identified studies suffered one or more serious methodological problems including the widespread practice of misclassifying former drinkers as abstainers. Our study was unable to incorporate the recommendation from Liang et al. [77] that former drinkers should in fact be included within the category of current drinkers according to previous drinking level so as to create an unbiased estimate of the risk relationship as only two eligible studies provided the risk estimates of differential drinking levels among former drinkers [6, 36]. Finally, as highlighted by Zeisser et al. [19], many studies classified occasional drinkers as low or medium volume drinkers creating the possibility of “reverse occasional drinker bias”. This may have differential effects according to gender but in the present study male occasional drinkers mostly had the lowest level of risk of prostate cancer. If occasional drinkers are included with low volume drinkers this could also have the effect of minimizing risk estimates. No studies were identified that were free of all possible types of misclassification error (ie when reverse occasional drinker bias is considered).

## Conclusions

In summary, the RR of prostate cancer morbidity or mortality significantly increased at low volume alcohol consumption levels (>1.3 g and <24g per day) compared to abstinence and a statistically significant a dose–response relationship was observed for the first time in a meta–analysis. The level of increased risk observed for low volume drinkers was relatively modest in the pooled analysis (8%), but was as high as 23% in the studies free of misclassification error. In either scenario, with a condition as prevalent as prostate cancer in developed countries, the public health implications of the findings are significant, so we suggest these are practically significant levels of risk for prevention purposes. Different forms of misclassification bias may differentially affect risk estimates, particularly the common practice of including former drinkers in the abstainer reference group may reduce risk estimates. Prostate cancer may need to be incorporated in future estimates of the burden of disease alongside other cancers (e.g. breast, oesophagus, colon, liver) and be integrated into public health strategies for reducing alcohol related disease. We recommend that future prospective studies on alcohol and disease seek to avoid biasing risk estimates by misclassifying either (i) former or occasional drinkers as abstainers, or (ii) occasional drinkers as low volume drinkers. Following Liang and Chikritzhs [77], we also recommend that former drinkers are classified with drinkers according to their past level of consumption.

## Additional files

Additional file 1: Meta-analysis data on prostate cancer and alcohol consumption. (XLSX 42 kb)
Additional file 2: Weighted RR estimates according to study characteristics. (DOCX 44 kb)
