# Supplementary material for: Is alcohol consumption a risk factor for prostate cancer? A systematic review and meta–analysis
Source: BMC Cancer. 2016 Nov 15;16:845. doi: 10.1186/s12885-016-2891-z (PMC5109713; doi:10.1186/s12885-016-2891-z)
Supplement: Additional file 2: — Weighted RR estimates according to study characteristics. (DOCX 44 kb) [file 12885_2016_2891_MOESM2_ESM.docx]

**Additional file 2. Weighted RR estimates according to study characteristics**

| Table A1. Weighted mean RR estimates of prostate cancer morbidity or mortality for any drinking versus “abstainers’ by study characteristics | | | | | |
| --- | --- | --- | --- | --- | --- |
| Study characteristics | N/n † | Any drink vs “abstainer” ‡ | | | |
|  |  | t–test P^1^ | RR | 95% CI | t–test P^2^ |
| Design |  |  |  |  |  |
| Cohort or case–cohort design | 17/72 | 0.2082 | 1.08 | 1.06 – 1.10 | 0.0001 |
| Hospital case–control design | 5/26 | ref | 1.01 | 0.92 – 1.12 | 0.7653 |
| Population case–control design | 5/28 | 0.0772 | 1.13 | 1.05 – 1.21 | 0.0010 |
| Outcome |  |  |  |  |  |
| Morbidity | 14/71 | 0.5018 | 1.10 | 1.05 – 1.15 | 0.0001 |
| Morbidity or mortality | 13/55 |  | 1.08 | 1.05 – 1.10 | 0.0001 |
| Country where a study conducted |  |  |  |  |  |
| US | 12/60 | 0.0005 | 1.10 | 1.08 – 1.13 | 0.0001 |
| Others | 15/66 |  | 1.02 | 0.98 – 1.06 | 0.3595 |
| Drink method |  |  |  |  |  |
| Adequate | 12/63 | 0.4374 | 1.10 | 1.05 – 1.16 | 0.0003 |
| Inadequate | 15/63 |  | 1.08 | 1.05 – 1.10 | 0.0001 |
| Mean age of study population ∏ |  |  |  |  |  |
| Mean=60.1, range: 41.4-70.0 | 27/126 |  | 1.02 | 0.97 – 1.07 | 0.4718 |
| 41.4–<61 | 16/79 | 0.7306 | 1.08 | 1.05 – 1.12 | 0.0001 |
| 61–70 | 11/47 |  | 1.08 | 1.04 – 1.11 | 0.0001 |
| Date at baseline (year) |  |  |  |  |  |
| 1967–1989 | 15/71 | 0.8970 | 1.08 | 1.04 – 1.12 | 0.0001 |
| 1990–2001 | 12/55 |  | 1.08 | 1.05 – 1.11 | 0.0001 |
| Exclude subject with history of cancer |  |  |  |  |  |
| Yes | 13/58 | 0.7354 | 1.08 | 1.06 – 1.11 | 0.0001 |
| No | 14/68 |  | 1.07 | 1.03 – 1.12 | 0.0024 |
| Study controlled for social status |  |  |  |  |  |
| No | 17/73 | 0.2881 | 1.07 | 1.03 – 1.10 | 0.0001 |
| Yes | 10/53 |  | 1.09 | 1.06 – 1.12 | 0.0001 |
| Study controlled for race |  |  |  |  |  |
| No | 20/88 | 0.0807 | 1.05 | 1.02 – 1.09 | 0.0052 |
| Yes | 7/38 |  | 1.10 | 1.07 – 1.12 | 0.0001 |
| Study controlled for smoking |  |  |  |  |  |
| Yes | 18/87 | 0.2195 | 1.06 | 1.03 – 1.10 | 0.0002 |
| No | 9/39 |  | 1.09 | 1.06 – 1.12 | 0.0001 |
| Misclassification biases |  |  |  |  |  |
| Both former & occasional biases | 10/41 | 0.5309 | 1.09 | 1.04 – 1.16 | 0.0014 |
| Former drinker bias | 6/26 | ref | 1.07 | 1.05 – 1.10 | 0.0001 |
| Occasional drinker bias | 5/28 | 0.2604 | 1.13 | 1.04 – 1·22 | 0.0038 |
| No biases | 6/31 | 0.8494 | 1.08 | 1.02 – 1.14 | 0.0077 |
| Note: † N= Number of studies and n= Number of risk estimates. ‡ Weights were calculated using the inverse of variance of natural log–RR. t–test P^1^: difference in the weighted RR between subgroups. t–test P^2^: Tests whether the weighted RR in each subgroup is significantly higher or lower than one. ∏ The RR increased by 2% as age increased by 10 years. | | | | | |

| Table A2. Weighted mean RR estimates of prostate cancer morbidity or mortality for low volume drinkers (≥1.3, <25 g/day) versus “abstainers’ by study characteristics | | | | | | | |
| --- | --- | --- | --- | --- | --- | --- | --- |
| Study characteristics | N/n † | |  | Low volume drinker vs “abstainer” | | | |
|  |  |  |  | t–test P^1^ | RR ‡ | 95% CI | t–test P^2^ |
| Design |  |  |  |  |  |  |  |
| Cohort or case–cohort design | 17/39 |  |  | 0.5299 | 1.07 | 1.04 – 1.10 | 0.0001 |
| Hospital case–control design | 5/9 |  |  |  | 1.02 | 0.88 – 1.18 | 0.7879 |
| Population case–control design | 5/14 |  |  | 0.4089 | 1.10 | 0.99 – 1.21 | 0·0627 |
| Outcome |  |  |  |  |  |  |  |
| Morbidity | 14/31 |  |  | 0.3024 | 1.10 | 1.03 – 1.17 | 0.0030 |
| Morbidity or mortality | 13/31 |  |  |  | 1.06 | 1.03 – 1.09 | 0.0003 |
| Country where a study conducted |  |  |  |  |  |  |  |
| US | 12/26 |  |  | 0.1432 | 1.08 | 1.05 – 1.12 | 0.0001 |
| Others | 15/36 |  |  |  | 1.04 | 0.98 – 1.09 | 0.1710 |
| Drink method |  |  |  |  |  |  |  |
| Adequate | 12/27 |  |  | 0.6015 | 1.09 | 1.01 – 1.17 | 0.0223 |
| Inadequate | 15/35 |  |  |  | 1.07 | 1.03 – 1.10 | 0.0001 |
| Mean age of study population ∏ |  |  |  |  |  |  |  |
| Mean=60.1, range: 41.4-70.0 | 27/62 |  |  |  | 1.02 | 0.96 – 1.08 | 0.5493 |
| 41.4–<61 | 16/35 |  |  | 0.7306 | 1.06 | 1.02 – 1.10 | 0.0074 |
| 61–70 | 11/27 |  |  |  | 1.08 | 1.04 – 1.13 | 0.0001 |
| Date at baseline (year) |  |  |  |  |  |  |  |
| 1967–1989 | 15/34 |  |  | 0.7632 | 1.07 | 1.03 – 1.12 | 0.0015 |
| 1990–2001 | 12/28 |  |  |  | 1.07 | 1.03 – 1.10 | 0.0010 |
| Exclude subject with history of cancer |  |  |  |  |  |  |  |
| Yes | 13/33 |  |  | 0.7826 | 1.07 | 1.03 – 1.10 | 0.0001 |
| No | 14/29 |  |  |  | 1.08 | 1.01 – 1.15 | 0.0212 |
| Study controlled for social status |  |  |  |  |  |  |  |
| No | 17/42 |  |  | 0.5468 | 1.08 | 1.04 – 1.12 | 0.0002 |
| Yes | 10/20 |  |  |  | 1.06 | 1.02 – 1.10 | 0.0075 |
| Study controlled for race |  |  |  |  |  |  |  |
| No | 20/47 |  |  | 0.9098 | 1.07 | 1.02 – 1.12 | 0.0041 |
| Yes | 7/15 |  |  |  | 1.07 | 1.03 – 1.11 | 0.0004 |
| Study controlled for smoking |  |  |  |  |  |  |  |
| Yes | 18/46 |  |  | 0.6791 | 1.07 | 1.03 – 1.12 | 0.0004 |
| No | 9/16 |  |  |  | 1.06 | 1.02 – 1.11 | 0.0041 |
| Misclassification biases |  |  |  |  |  |  |  |
| Both former & occasional biases | 10/20 |  |  | 0.1883 | 1.10 | 1.03 – 1.18 | 0.0077 |
| Former drinker bias | 6/16 |  |  | ref | 1.05 | 1.01 – 1.08 | 0.0135 |
| Occasional drinker bias | 5/10 |  |  | 0.5969 | 1.08 | 0.95 – 1.24 | 0.2205 |
| No biases | 6/16 |  |  | 0.0767 | 1.12 | 1.05 – 1.19 | 0·0009 |
| Note: † N= Number of studies and n= Number of risk estimates. ‡ Weights were calculated using the inverse of variance of natural log–RR. t–test P^1^: difference in the weighted RR between subgroups. t–test P^2^: The weighted RR in each subgroup is significantly higher or lower than one. ∏ The RR increased by 2% as age increased by 10 years. | | | | | | | |
